# RNA CoSSMos 2.0: an improved searchable database of secondary structure motifs in RNA three-dimensional structures

**DOI:** 10.1093/database/baz153

**Published:** 2020-01-16

**Authors:** Katherine E Richardson, Charles C Kirkpatrick, Brent M Znosko

**Affiliations:** Saint Louis University, Department of Chemistry, 3501 Laclede Avenue, St. Louis, MO 63103 USA

## Abstract

The RNA Characterization of Secondary Structure Motifs, RNA CoSSMos, database is a freely accessible online database that allows users to identify secondary structure motifs among RNA 3D structures and explore their structural features. RNA CoSSMos 2.0 now requires two closing base pairs for all RNA loop motifs to create a less redundant database of secondary structures. Furthermore, RNA CoSSMos 2.0 represents an upgraded database with new features that summarize search findings and aid in the search for 3D structural patterns among RNA secondary structure motifs. Previously, users were limited to viewing search results individually, with no built-in tools to compare search results. RNA CoSSMos 2.0 provides two new features, allowing users to summarize, analyze and compare their search result findings. A function has been added to the website that calculates the average and representative structures of the search results. Additionally, users can now view a summary page of their search results that reports percentages of each structural feature found, including sugar pucker, glycosidic linkage, hydrogen bonding patterns and stacking interactions. Other upgrades include a newly embedded NGL structural viewer, the option to download the clipped structure coordinates in *.pdb format and improved NMR structure results. RNA CoSSMos 2.0 is no longer simply a search engine for a structure database; it now has the capability of analyzing, comparing and summarizing search results.

Database URL: http://rnacossmos.com

## Introduction

A deep understanding of the primary, secondary and tertiary structures of RNA and the relationship between these levels of structure is essential to understand and predict how RNA folds, gain knowledge of its biological functions and design potential therapeutics. A variety of approaches have been taken in predicting RNA 3D structure from sequence, with template-based approaches resulting in a particularly high level of accuracy (1). A template-based approach uses solved RNA 3D structures or fragments as templates to build an initial structure of the structure to be predicted. A critical area for improvement of RNA 3D structure prediction is the prediction of non-Watson–Crick interactions, such as those found in loops (1). Therefore, the utility of a database that contains 3D structure templates for secondary structure motifs remains high. Herein, we use the term ‘motif’ to describe a secondary structure element, such as an internal loop, bulge loop or hairpin loop. This definition of a secondary structure motif does not necessarily imply sequence similarity, 3D structural similarity nor a shared biological function.

Currently, there are several databases that contain 3D structures for secondary structure motifs. The Structural Classification of RNA (SCOR) database was created through a manual classification and inspection of RNA structures (2, 3). The SCOR database provides 3D structures for hairpin and internal loops deposited in the Protein Data Bank (PDB) before 15 May 2003. RNA Fragments Search Engine and Database (RNA FRABASE) 2.0 (4, 5), which is updated monthly, converts PDB files of RNA structures to a dot-bracket notation using the RNAView program (6) to identify base pairs. RNA FRABASE 2.0 allows users to search for RNA 3D structure fragments using the user input sequence and dot-bracket secondary structure notation (4, 5). The RNA 3D Motif Atlas (7) uses Find RNA 3D (FR3D) (8) to search for hairpin and internal loops and classify the loops into 3D structure motifs. FR3D uses a base-centered approach in which each base is represented by a single center point to compare RNA 3D structures (8). The RNA Characterization of Secondary Structure Motifs (RNA CoSSMos) database was first released in 2011 with weekly automatic updates (9). To create the RNA CoSSMos database, the RNA-containing 3D structures in the PDB (10) were searched for internal, bulge and hairpin loops using *MC-Search* (11, 12). *MC-Search* uses user-defined base pairing interactions and subgraph isomorphism algorithms to search PDB files for RNA secondary structure motifs (11, 12). Subsequently, *MC-Annotate* (11, 12) was used to determine structural information including sugar puckers, glycosidic linkages, hydrogen bonding patterns and stacking interactions within the loop and closing base pair. *MC-Annotate* uses homogenous transformation matrices to calculate base–base interactions (11, 12). The results of *MC-Search* and *MC-Annotate* were pre-compiled into an online database for fast retrieval with an intuitive user-interface. RNA CoSSMos was designed to complement similar databases previously created. What continues to make the RNA CoSSMos database unique is the intuitive graphical user interface that does not require knowledge of a specialized syntax and the detailed structural annotation provided by *MC-Annotate* that includes not only base-pairing interactions but additional structural information including sugar puckers, glycoside linkages and stacking interactions. The option to search the database using any combination of PDB information, experimental parameters, motif and/or sequence allows users to highly customize their database viewing.

Since the RNA CoSSMos release in 2011, the RNA-containing structures in the PDB have increased from 2156 to over 3909, leading to subsequent growth of the RNA CoSSMos database. An RNA CoSSMos search returns results which can be viewed individually on the website. For searches that return a large number of results, individual viewing can become a daunting task. Additionally, studies that search for tertiary structural patterns among secondary structure motifs, such as those performed on single mismatches (13), have shown the need for users to be able to further analyze and summarize their search results. RNA CoSSMos 2.0 is no longer simply a search engine for a structure database; it now has the capability of analyzing, comparing and summarizing the search results. The two main improvements to the RNA CoSSMos database are new functionalities to (i) calculate an average and representative structure of the results and (ii) provide a summary page with percentages of structural features among the results. Additional improvements include (iii) the addition of a second closing base pair requirement for loop motifs, (iv) the replacement of Jmol by NGL Viewer for 3D structure viewing (14–17), (v) the option to download the coordinates of the clipped structures in ^*^.pdb format and (vi) the addition of the model numbers of NMR results to the database. The RNA CoSSMos website has been moved to a faster webhost and is now available at http://rnacossmos.com.

## New features and improved content

### New results analysis features

In addition to viewing search results individually, users can now further explore their search results in the ‘Summarize Results’ tab. Two features have been added to the website to allow users to obtain a summary of their search results: (i) an average structure feature and (ii) a results summary feature. These features (described below) can be chosen together or independently, depending on the needs of the user. Users can further customize their summary by selecting which structures in their search results should be included in the summary calculation. If the user’s search contains ≤350 results, they can select the desired results using check boxes. Alternatively, the user can download the search results as a delimited spreadsheet, edit the file to select for results to include in the summary and then upload the file to the RNA CoSSMos website. An option is also available to include all of the search results in the summary calculations.

#### Average structure.

**Figure 1:**
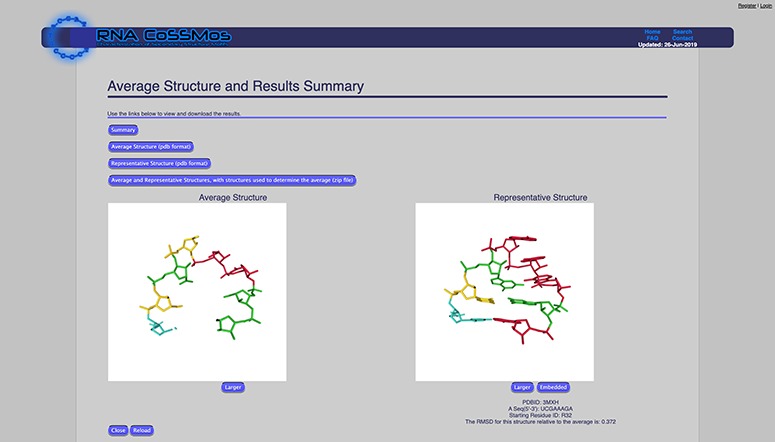
The RNA CoSSMos average structure page showing the average and representative structure for all GNRA tetraloops. A total of 4724 GNRA tetraloops were included in the calculations using the backbone and three atoms from each base.

The average structure feature calculates an average structure of all of the selected search results using a Python script. Users can customize this calculation by selecting which atom coordinates they would like included in the calculation. Atom coordinate options are (i) phosphate atoms only, (ii) phosphate-sugar backbone atoms or (iii) phosphate-sugar backbone and bases. If users select the ‘phosphate-sugar backbone and bases’ option, all of the atom coordinates from the backbone and three atom coordinates from each base (N9, C8 and C4 for purines and N1, C2 and C6 for pyrimidines) are used in the calculation to allow users to calculate average structures for results with differing sequences. To calculate the average structure, the coordinates are superimposed using the Kabsch algorithm (18) to calculate the optimal rotation matrix. The average structure (which consists of the selected coordinates) is available for viewing on the website, and the coordinates can be downloaded as a ^*^.pdb file. In addition to the average structure, the root mean square deviation (RMSD) of each result structure compared to the average structure is calculated, and the result that has the smallest RMSD to the average structure is reported as the representative structure. The representative structure can also be viewed on the website and downloaded as a ^*^.pdb file (Figure 1).

#### Results summary.

**Figure 2:**
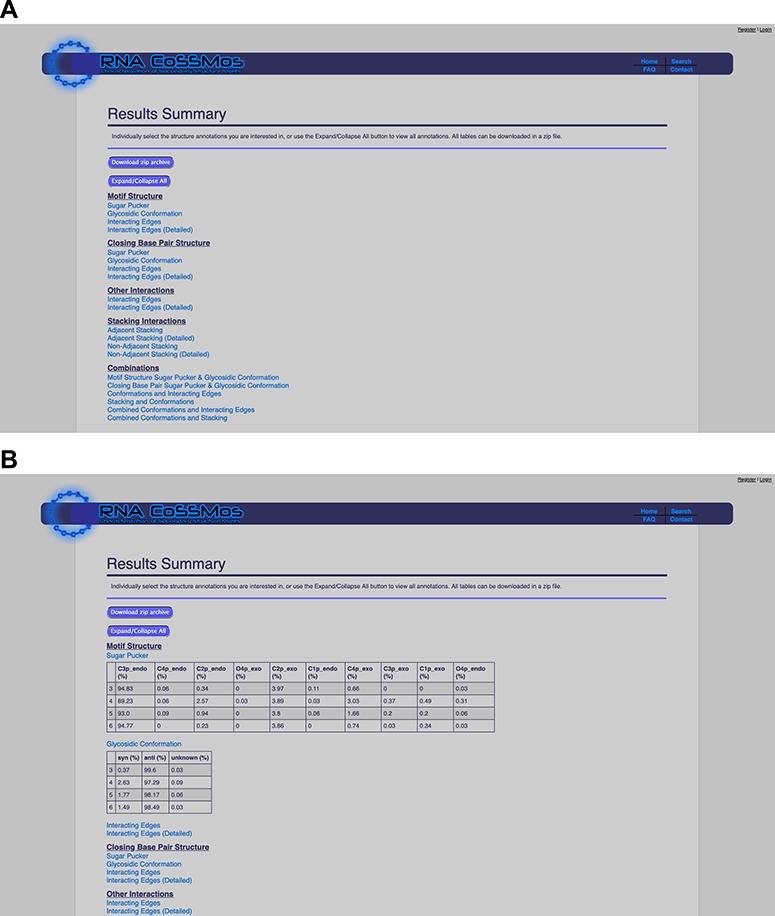
The RNA CoSSMos results summary page (**A**) collapsed and (**B**) expanded for ‘Sugar Pucker’ and ‘Glycosidic Conformation’.

The results summary compiles all the individual *MC-Annotate* results into tables that report percentages of sugar puckers, glycosidic linkages, hydrogen bonding patterns and stacking interactions within the loop and closing base pairs across all of the selected RNA CoSSMos search results. These percentages are reported for individual features (e.g. the sugar pucker of the first base in the loop) and combinations of structural features (e.g. the residue conformations of the first and last bases in the loop and hydrogen-bonding interactions between these bases). The tables utilize the residue numbering system described in this paper and on the RNA CoSSMos FAQ wiki page (http://rnacossmos.com/faq2.php). For user convenience, the tables can be expanded and collapsed so that users can focus on the structural annotations of greatest interest (Figure 2). Additionally, all of the tables can be downloaded as a zipped file that contains individual delimited spreadsheets for each table.

### New requirement for two closing base pairs

**Figure 3:**
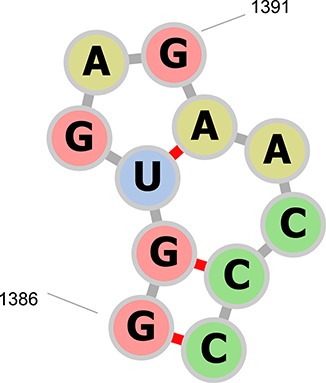
The secondary structure representation of residues A1386–A1396 in PDB ID 1N8R drawn with forna (24). The previous RNA CoSSMos database contained a hairpin of seven nucleotides in residues A1387–A1395, a hairpin of three nucleotides in residues A1388–A1392, and a bulge of two nucleotides in residues A1387–A1388 and A1392–A1395. RNA CoSSMos 2.0 only contains a hairpin of seven nucleotides in residues 1386–1396.

Extensive use of the previous RNA CoSSMos database returned results containing substantial overlap between loop motifs as well as loop instances with one or no closing base pair. For example, residues A1386–A1396 in the structure of the large ribosomal subunit in complex with virginiamycin M (PDB ID 1N8R) contained a hairpin of seven nucleotides in residues A1387–A1395, a hairpin of three nucleotides in residues A1388–A1392 and a bulge of two nucleotides in residues A1387–A1388 and A1392–A1395 (Figure 3). To reduce the overlap and improve the integrity of the database, a new set of *MC-Search* input descriptors were written that require two A-U, C-G, G-C, G-U, U-A or U-G closing base pairs. The requirement of two closing base pairs for secondary structures has been shown to improve the accuracy of RNA folding (19). With these new input descriptors, residues A1386–A1396 in PDB ID 1N8R will only contain a hairpin of seven nucleotides in the RNA CoSSMos 2.0 database.

#### Residue numbering system.

**Figure 4:**
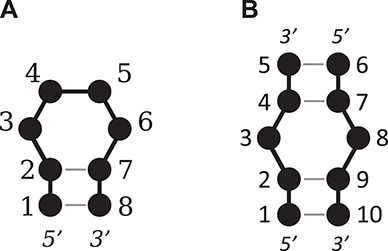
An illustration of the numbering system used for (**A**) a tetraloop and (**B**) a 1 × 1 nucleotide internal loop.

In order to compare the structures and structural features of the results, a common residue numbering system was necessary. The residue ID numbers within each result were renumbered as follows (Figure 4). For hairpin loops, the second 5′ closing base is assigned as the first base, with the remaining bases assigned sequentially from the 5′ to the 3′ end. For internal and bulge loops, the second 5′ closing base of the ‘A’ strand or ‘top’ strand is assigned as the first base, with the remaining bases in this strand assigned sequentially from the 5′ to the 3′ end. The second 5′ closing base of the ‘B’ strand or ‘bottom’ strand is assigned the next position where the ‘A’ strand terminated, then the remaining bases in the ‘B’ strand are sequentially assigned from the 5′ to 3′ end.

### Improved structure viewing

RNA CoSSMos previously utilized a Jmol applet (20) for 3D structure viewing. For RNA CoSSMos 2.0, the Jmol applet has been replaced by NGL Viewer (http://nglviewer.org) which does not require the use of Java or plugins. The NGL molecular viewer has replaced the Jmol viewer on the individual detailed results pages and is also utilized to view the average and representative structures. Previously, users could download a snapshot of the Jmol viewer. Now, users can download a clipped ^*^.pdb file of the motif structure in which the residue ID numbers have been renumbered according to the residue numbering system described in this paper. This will allow users to view and manipulate the clipped structures on their own computers using their preferred software in addition to 3D structure viewing on the RNA CoSSMos website. Additional viewing options allow users to see individual motifs and representative structures embedded within the global structure found in the ^*^.pdb file.

### Improved NMR structure handling

Model numbers corresponding to those in the original PDB structure have been added to the RNA CoSSMos 2.0 database for ensemble structures solved by NMR. For each NMR ensemble within each motif, the model that is closest to the average of the ensemble results is selected as the representative model for the ensemble. The representative models are denoted in the database with an ‘*’ after the model number. Users can view the detailed results of the individual models or can utilize the new summary features to learn about structural information within the ensemble or across multiple selected ensembles. When a user wishes to utilize the ‘Summarize Results’ tab with results that contain NMR structures, the user is given the option to include all of the models within the ensemble or only the representative model in the summary calculations.

## Example utilization

Hairpins that contain four bases in the loop are commonly referred to as tetraloops. Tetraloops with the sequence GNRA (N = any nucleotide, R = A or G) often form an unusually stable 3D fold that is well-documented (21, 22). To demonstrate the usefulness of the new analysis features of the RNA CoSSMos database, we conducted a search for tetraloops with the GNRA loop sequence. The RNA CoSSMos database currently contains 4724 tetraloops with the GNRA loop sequence. Utilizing the original version of the RNA CoSSMos database, users could then explore the detailed results pages of the 4724 results individually on the website or by downloading a tab-delimited file that could be explored using spreadsheet software on their computer. There were no tools available on CoSSMos to compare structures or summarize results. Now, using the updated RNA CoSSMos database, users can compare and/or summarize the results using the ‘Average Structure’ and ‘Results Summary’ features. The average and representative structures of the 4724 tetraloops were calculated using the backbone and three atoms from each base (Figure 1). These structures can be viewed on the website or can be downloaded as templates for RNA structure prediction. Furthermore, the structural features of all 4724 tetraloops were tabulated using the ‘Results Summary’ feature, showing that approximately 90, 85 and 95% of the GNRA tetraloops contain hydrogen bonding between the G nucleobase and A nucleobase, base stacking of N on R, and base stacking of R on A, respectively, three features characteristic of the GNRA fold (23). A similar analysis utilizing the new features has identified three new tetraloop sequence families (25). We anticipate that the new features can be utilized for a variety of similar and novel purposes.

## Conclusions and future directions

The new RNA CoSSMos database continues to provide users with a unique online tool to search for 3D structure characteristics of RNA secondary structure motifs using a simple and intuitive graphical user interface but now allows its users to explore and compare structural features and patterns among their customized set of search results in addition to viewing the results individually. Additional updates not described here to improve ease of use, user customization and database completeness and accuracy have been made to ensure that the database remains a reliable and valuable tool for the scientific community. In 2014, the PDBx/mmCIF file format became the standard PDB format to allow for large structures that cannot be represented in the *.pdb file format. Currently, ~6% of RNA containing structures are only offered in the PDBx/mmCIF file format. Future versions of the RNA CoSSMos database will incorporate the PBDX/mmCIF file format, and future directions of the database will be driven by feedback from the users. As the PDB continues to grow, so will the RNA CoSSMos database and its capabilities.

### Availability

The RNA CoSSMos 2.0 database is freely available online and is now hosted at a new URL http://rnacossmos.com. We will maintain the redirection from the previously used URL (http://cossmos.slu.edu) for the foreseeable future, but users are encouraged to update their links accordingly.
